# COMPARISONS BETWEEN COGNITIVELY-IMPAIRED AND HEALTHY OLDER ADULTS ON NAVIGATION METRICS

**DOI:** 10.1093/geroni/igad104.3242

**Published:** 2023-12-21

**Authors:** Michael Pervratil, Dorota Kossowska-Kuhn, Neil Charness

**Affiliations:** Florida State University, Tallahassee, Florida, United States; Florida State University, Tallahassee, Florida, United States; Florida State University, Tallahassee, Florida, United States

## Abstract

Being able to navigate one’s environment is a critical skill that contributes to daily living in a variety of circumstances. Navigation is also a complex skill, composed of multiple subskills that play a part in overall performance. Along with other facets of cognition, navigation ability is expected to decline as a part of normal aging processes. What is of additional interest is how cognitive impairment (CI) relates to navigation in older adults. Throughout the literature, differences in navigation have been demonstrated between CI populations and healthy older adults. The current study sought to probe these components of navigation by comparing CI and healthy older adults on several measures of navigation ability. A sample of CI older adults were collected across three sites in the eastern United States. As a part of a larger project, participants completed objective and subjective measures of navigation, and their results were compared with a large sample of community-dwelling older adults (ages 60-90). The goal of these analyses was to determine where CI and healthy older adults differed in navigation. The CI sample performed significantly worse on the spatial orientation test (SOT) and reported significantly worse outcomes on a general wayfinding scale. However, there was no significant difference in self-assessed need for help when navigating or frequency of feeling lost. This divergence in outcomes is highlighted and deliberated further. Utilization of subjective and objective measures is discussed along with limitations of the data.

